# Crystal Morphology and Associated Face-Specific Growth Kinetics of Tolfenamic Acid as a Function of Its Solution Crystallization Environment[Fn fn1]

**DOI:** 10.1021/acs.cgd.5c01129

**Published:** 2025-10-21

**Authors:** Yu Liu, Cai Y. Ma, Junbo Gong, Kevin J. Roberts

**Affiliations:** † School of Chemical Engineering, Shenyang University of Chemical Technology, Shenyang 110142, China; ‡ Centre for the Digital Design of Drug Products, School of Chemical and Process Engineering, 4468 University of Leeds, Woodhouse Lane, Leeds LS2 9JT, U.K.; ∥ State Key Laboratory of Chemical Engineering, 12605 Tianjin University, Tianjin 300072,China

## Abstract

The crystal morphology and face-specific growth kinetics of tolfenamic acid (TFA) forms I and II are investigated through an integrated molecular modeling and experimental approach. Morphology predictions based on attachment energy calculations are consistent with experimental observations, with needle-like habits for both forms, although with some subtle differences in the capping faces formed, which can be attributed to the variations in intermolecular packing and surface chemistry. The solvent polarity is found to significantly influence the crystal growth of both forms: for instance, polar solvents, such as ethanol, promote higher aspect ratios by disrupting hydrogen bonding at prismatic faces, while nonpolar solvents, such as toluene, are found to hinder elongation of the crystal habit by providing strong solute/solvent aromatic stacking interactions at the capping faces. Examination of the measured growth rates for form I in ethanolic solutions reveals markedly slower growth rates (0–0.02 μm/s) on the prismatic faces (e.g., {0 1 1}) when compared to the capping faces e.g., {1 0 0} (0.044–0.555 μm/s), consistent with the lower surface intermolecular unsaturation and limited solute binding on the former faces. Examination shows that the facet crystal growth rates of form II (at a supersaturation of 0.3) are higher than that for form I for both capping and prismatic faces, consistent with the ease of crystallization of form II in ethanolic solutions. Analysis of the growth rate data for form I as a function of supersaturation reveals a good fit using a BCF model, with the surface integration at the crystal/solution interface rather than solute mass transfer in the bulk solution being identified as the rate-limiting step for the prismatic faces. This is in contrast to the capping faces, which is found to be less well-defined with mass transfer and surface integration being more balanced depending on the degree of solution supersaturation. The interplay between solvent-dependent surface interactions and intermolecular packing with the crystal face-specific growth kinetics is highlighted, contributing well toward the development of a predictive framework for the design and control of the solid-form properties of organic materials.

## Graphical abstract

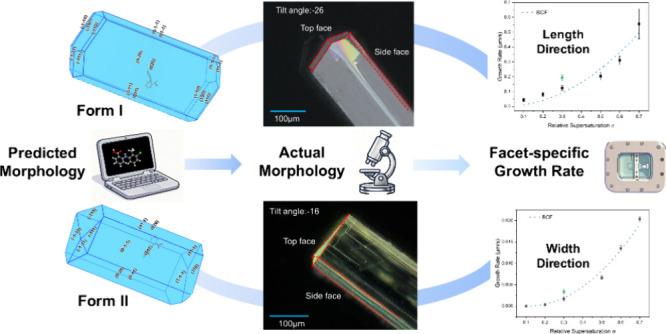

## 1 Introduction

Crystallization is an important purification and separation unit operation for the fine chemical process industries in the production of high-value organic products. Due to the anisotropic nature of organic materials, the crystalline products can exhibit diverse anisotropic morphologies and associated surface properties, which can directly impact the physicochemical properties of the products formed, such as stability, hygroscopicity, filtration efficiency, purity, and tableting properties.^1^ Therefore, gaining an understanding at the fundamental level of the mechanisms governing the crystal morphology and its associated face (*hkl*)-specific growth kinetics can be important in order to obtain crystalline products that have predesired properties.

Crystal morphology is predominantly dependent on the intermolecular packing in the solid state and, in particular, how these interactions are terminated at crystal surfaces. In principle, crystal morphology and physicochemical properties of crystalline particles can be predicted based on the bulk crystallographic structural data. Many efforts have been made in this field,^2^ such as using synthonic engineering approaches to predict the intermolecular interactions (synthons),^3^ relative growth rates, and crystal morphology,^4–7^ using software such as HABIT98.^8,9^ However, the experimentally observed crystal morphology can also be modified by the molecular nature of the solution environment, particularly when solvent-facet interactions associated with solute desolvation during the crystallization processes act in a habit face (*hkl*)-specific manner and hence alter the surface energetics and hence the dynamics of the crystal growth process.^10^ Understanding such effects can be assessed through molecular-scale modeling, aiming to characterize the surface chemistry of the individual crystal habit facet surfaces, particularly their propensity for solvent–surface interactions. For example, solvent-mediated morphological changes have been characterized in lovastatin,^6^ L-glutamic acid,^11^ aspirin,^12^ and the polymorphs of ritonavir.^13^ These have highlighted the interplay between solvent properties, crystal morphology, and the structural chemistry of the crystal surfaces under various growth environments. Despite this progress, fundamental studies on morphology and associated growth kinetics for the different crystal habit faces of organic materials, and especially comparative studies for different crystalline polymorphic forms, remain quite limited.

Tolfenamic acid (TFA) is a nonsteroidal anti-inflammatory drug with nine reported conformational polymorphs.^14,15^ Of these, the most encountered polymorphs have been found to be the stable form I^14^ and metastable form II.^14^ Although both forms have been found to adopt quite similar needle-like crystal morphologies, the detailed differences between their morphologies and solvent-dependent growth behaviors remain quite poorly understood. Moreover, it is noticeable that forms I and II can also be crystallized concomitantly,^16,17^ with previous studies revealing the concomitant polymorphism mechanism from a solubility and nucleation perspective.^17,18^ The concomitant crystallization behavior may also be related to the competitive nucleation and growth process; hence, further investigation of the growth kinetics would be of significant importance. TFA thus serves as a good model for crystal growth research, but despite this, future work is still needed on characterizing its morphology and face-specific growth kinetics.

In this work, an integrated study encompassing both molecular modeling and experimental work has been carried out, aiming to investigate the morphology and crystal growth kinetics of TFA forms I and II together with an assessment of the surface chemistry and solvent–surface intermolecular interactions in order to rationalize solvent-driven morphological variations. Through this, this research seeks to reveal a fundamental understanding of the TFA crystal growth process and also provide a contribution to the wider predictive framework for optimizing crystal morphology in organic materials to meet product requirements by facilitating tailored solvent mediation of the growth environments and process conditions.

## 2 Materials and Methods

### 2.1 Materials

TFA (>99%) was purchased from Fluorochem Ltd. All solvents were obtained from ThermoFisher and were of analytical grade. All chemicals were used directly without further purification. The crystal structures of TFA form I (ref code: KAXXAI01)^14^ and form II (ref code: KAXXAI)^14^ were obtained from the Cambridge Structural Database.^19^

### 2.2 Experimental Methods

#### 2.2.1 Preparation of TFA Forms I and II

TFA was recrystallized to prepare the two polymorphic forms I and II of TFA. Form I was obtained through slow cooling crystallization using 20 mL vessels, while form II was obtained through fast cooling crystallization.^18^ TFA solutions were first prepared at 50 °C with setting concentrations and held for 1h to obtain a clear solution. Then, the solutions were cooled using the two types of cooling rates (fast and slow) until crystals were obtained. Four solvents exhibiting different polarities and bonding motifs (acetonitrile (aprotic polar), methanol and ethanol (protic polar), and toluene (aprotic apolar)) were used in batch cooling crystallization experiments in order to compare the crystal morphologies of the two forms obtained in various solution environments. The concentrations for each solvent were determined according to their solubilities at 20 °C, e.g., 38 g kg^–1^ for ethanol, 21 g kg^–1^ for methanol, 1.5 g kg^–1^ for toluene, and 6.2 g kg^–1^ for acetonitrile.

#### 2.2.2 Solid-Form Characterization

Powder X-ray diffraction (PXRD) was performed for determining the polymorphic forms of two phases of TFA. The characterization was carried out using a Bruker D8 advanced X-ray diffractometer with Cu Kα radiation, using a scanning range of 4–40° (2θ) and a step time of 0.7 s per step. The color of the crystals was also used as a further aid to distinguish the two polymorphic forms of TFA (form II: yellow crystals; form I: white crystals). The morphologies of TFA crystals were characterized by a KEYENCE VHX7000 digital optical microscope, which has the facility for providing a tilted (±30°) optical view.^20^

#### 2.2.3 Growth Rate Measurements

##### 2.2.3.1 Experimental Setup

The growth rates for the {1 0 0} and {0 1 1} faces of single crystals as a function of the solution supersaturation were achieved using a temperature-controlled crystal growth cell. The system comprises a glass cuvette cell^4,5^ (Figure 1a), an optical polarizing microscope (Olympus BX51) integrated with a CCD Infinity camera, connected to a computer to capture images, and analysis software. A UV cuvette cell 0.5 mL (54 × 1 × 1 mm) was used as the crystallization vessel, which was submerged in a shallow cell of circulating water whose temperature was controlled by a Julabo F25 water bath.

**Figure 1:**
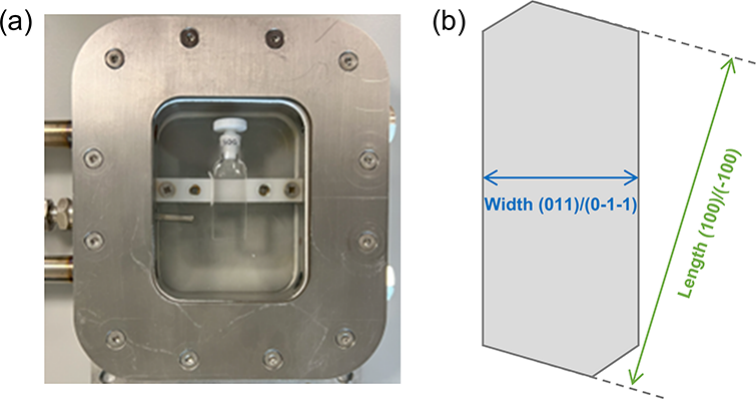
Image of the growth cell used for the growth experiment and schematic illustration of the determination of the growth rate of form I by measuring the distances between parallel crystal faces.

##### 2.2.3.2 Experimental Procedure

Solutions with different supersaturations (1.1–1.7 for 20 °C) were prepared first by dissolving the TFA solute in ethanol based on the published solubility data.^17,18^ The solutions prepared were then transferred to a cuvette cell using pipettes with the crystal seed of TFA also being added into the cuvette cell, which was then sealed rapidly and placed at the bottom of the growth cell (Figure 1a). The temperature of the growth cell was set to 55–60 °C (higher than the supersaturated temperature) to produce a slightly undersaturated solution for the slight dissolution of the single crystal seed, hence removing any possible imperfections at the crystal surface and also reducing the seed size to achieve the typical length (700–1000 μm) and width (30–40 μm). After that, the solution was cooled to 20 °C to generate supersaturated solutions (1.1–1.7) for the crystal growth rate experiments as a function of supersaturation.

##### 2.2.3.3 Data Acquisition

Two pairs of opposite faces of the TFA crystal were selected for growth rate analysis. Image analysis software was used to capture a sequence of crystal images at constant time intervals (ca. every 10 min) during the crystal growth process; hence, the distances between the parallel crystal faces were then determined as a function of time, as depicted in Figure 1b. The effect of facet inclination with respect to the observational plane were corrected based on the known interfacial angles between the habit planes. The ability of the microscope to tilt (±30°) enabled the morphological features, particularly the capping faces of TFA, to be well-characterized from the projected views as a function of the tilt angle.

The distances determined between the paired faces with respect to time were then used in the linear fitting of the data in order to calculate the facet growth rates as given in Figure S1 (Supporting Information (SI)). In some cases, two stages in the growth rate behavior were observed in the length vs time plot, as shown in Figure S2 (SI). The first stage follows a nearly linear relationship because the supersaturation remains relatively constant and close to its initial set value, while the growth rate gradually decreases in the second stage due to the consumption of solute concentration, hence the reduction of the solution supersaturation. Therefore, only the initial rate data were used for the determination of the growth rates (Figure S2 (SI)).

#### 2.2.4 Derivation of Growth Interface Kinetics

The growth kinetics were assessed through consideration of two core series process steps: first, the mass transfer (MT) diffusion of the growth unit from the bulk solution to the crystal surface and, second, the integration of solute growth units into the growing crystal habit surfaces (GS) of the crystal. The overall growth model^5,21^ used thus encompasses both of these two factors to fit the measured growth rate data in this work, as expressed in eq 1:

$$G\left(\frac{m}{s}\right)=\frac{1}{\frac{1}{k_{\mathrm{MT}}^{\prime }}+\frac{1}{k_{\mathrm{GS}}}}(\sigma )$$ 1

where *G* is the facet growth rate, $\frac{1}{k_{\mathrm{MT}}^{\prime }}$ is the resistance of mass transfer in the bulk solution, while $\frac{1}{k_{\mathrm{GS}}}$ is the resistance of integration of growth units at the surface. σ is the relative supersaturation, which can be calculated using eq 2:

$$\sigma =\frac{x}{x_{e}}-1$$ 2

where *x* is the solution concentration and *x*_*e*_ is the mole fraction solubility with *k*_GS_ being dependent on the mechanistic models:

For the power law model,

$$k_{\mathrm{GS}}=k_{G}(\sigma {)}^{r-1}$$ 3

For the B&S model,

$$k_{\mathrm{GS}}=k_{G}(\sigma {)}^{-1/6}\exp\left(\frac{A_{1}}{\sigma }\right)$$ 4

For the BCF model,

$$k_{\mathrm{GS}}=k_{G}(\sigma )\tanh\left(\frac{A_{2}}{\sigma }\right)$$ 5

where *k*_G_ is the growth rate constant, *r* is the growth exponent with *r* = 1 being consistent with a roughened growth interface interaction mechanism, and *A*_1_ and *A*_2_ are kinetic fitting parameters.

#### 2.2.5 Computational Molecular Modeling

##### 2.2.5.1 Intermolecular Interaction

The intermolecular pair interaction energies for the two TFA forms were calculated using HABIT98 ^8,9^ together with the Dreiding^22^ force field and the partial electronic changes calculated using MOPAC^23^ with the Austin Model 1 (AM1) approach. The calculated intermolecular potential energies (*E*) were subdivided into the constituent van der Waals (vdW), hydrogen bonded, and electrostatic energies^24^ of the interaction, as shown in eq 6:

$$E=\overset{M_{i}}{\underset{i=1}{\sum }}\overset{M_{j}}{\underset{j=1}{\sum }}\left[\left(-\frac{A_{ij}}{r_{ij}^{6}}+\frac{B_{ij}}{r_{ij}^{12}}\right)+\left(-\frac{C_{ij}}{r_{ij}^{10}}+\frac{D_{ij}}{r_{ij}^{12}}\right)+\frac{q_{i}q_{j}}{Dr_{ij}}\right]$$ 6

where *A*_*ij*_, *B*_*ij*_, *C*_*ij*_, and *D*_*ij*_ are atom–atom force field parameters for atoms *i* and *j* in the first and second molecules, respectively; *q*_*i*_ and *q*_*j*_ are atomic point charges; *D* is the dielectric parameter; and *r*_*ij*_ is the central distance between atoms *i* and *j*. Their overall 2D intermolecular arrangements were visualized using Material Studio.^25^ Through this, the bulk intermolecular interactions (intrinsic synthons) were characterized, classified, and ranked in terms of their interaction nature and strengths.

##### 2.2.5.2 Morphology Prediction

The crystal morphology was predicted using the attachment energy method^26^ using HABIT98.^8,26^ The main crystal faces expected to be within the overall morphology together with their growth layer thickness (*d*_*hkl*_) being determined by the BFDH method^26,27^ using Mercury.^28^ The dominant intermolecular interactions identified in Section 2.2.3.1 were partitioned between the intrinsic synthons, i.e., those fully coordinated within the growth layer or slice (*E*_sl_^*hkl*^) and the extrinsic synthons (*E*_att_^*hkl*^) whose interactions were surface-terminated by the facet planes of the external morphology of the crystal. Here, *E*_sl_^*hkl*^ is the slice energy that is associated with the stability of the surface, while *E*_att_^*hkl*^ is the surface attachment process that promotes crystal growth. The lattice energy (*E*_cr_) is the sum of *E*_sl_^*hkl*^ and *E*_att_^*hkl*^, as shown in eq 7.

$$E_{\mathrm{cr}}=E_{\mathrm{sl}}^{hkl}+E_{\mathrm{att}}^{hkl}$$ 7

The crystal facet-specific relative growth rates were assumed to be proportional to *E*_att_^hkl^ ^29^ with the overall predicted crystal resulting from a 3D polar plot of *E*_att_^hkl^ using a Wulff plot^30^ for each TFA form. The surface anisotropy factor (ξ_*hkl*_) was also calculated using eq 8, to represent the degree of synthon saturation for the different crystal surfaces.

$$\xi_{hkl}=\frac{E_{\mathrm{sl}}^{hkl}}{E_{\mathrm{cr}}}$$ 8

## 3 Results and Discussion

### 3.1 Analysis of the Bulk and Surface Structure of the Crystals

Figure 2 provides the intermolecular packing patterns for the TFA crystallographic structures as viewed along the a axis, highlighting the rather similar packing modes adopted for the two forms. Examination of the structures of both TFA polymorphs reveals the existence of hydrogen-bonded dimeric interactions between the carboxylic groups with these motifs governing the crystal chemistry in both forms I and II together with aromatic stacking and vdW interactions. The main significant difference between the crystal chemistry of the two forms reflects their different molecular conformations, which, in turn, impact their different intermolecular interaction strengths.

**Figure 2:**
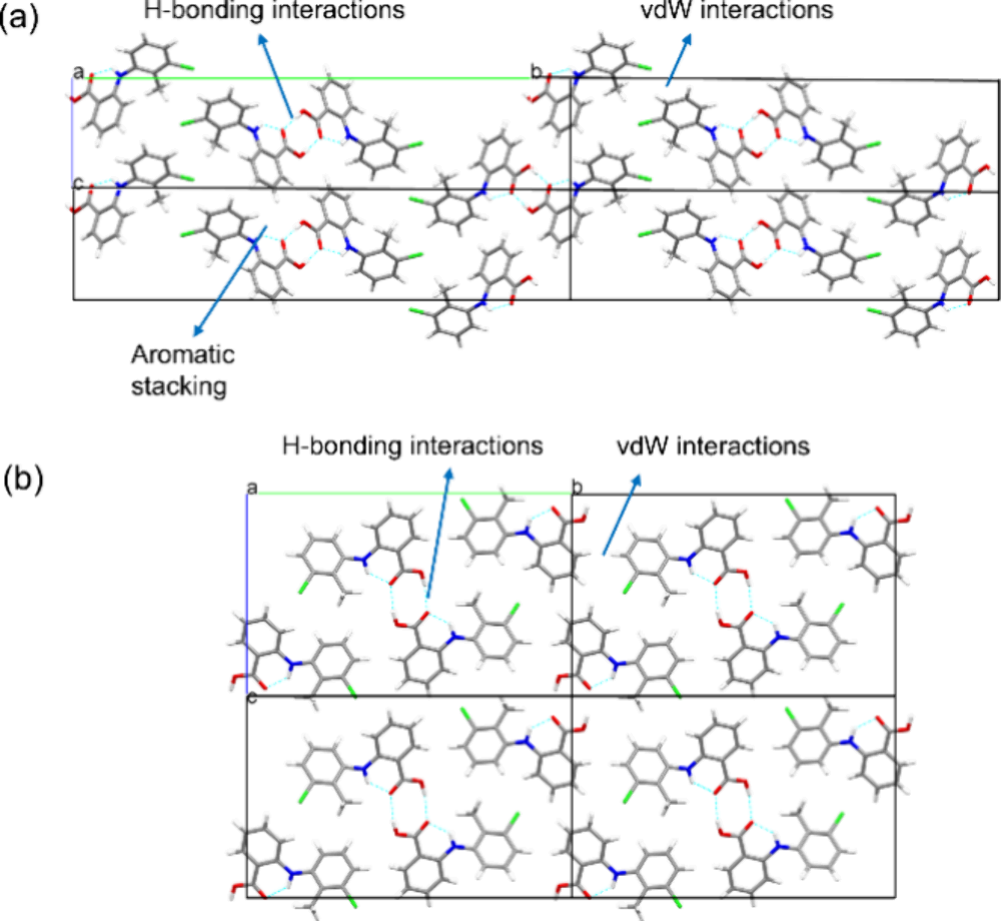
Intermolecular packing within the crystallographic unit cells for the tilted and planar polymorphs of forms I (a) and II (b), respectively.

Intrinsic synthon analysis reveals^18^ that hydrogen bonds contributed most to the stability of form I crystal structure, while aromatic stacking synthons were found to be the strongest synthons for form II, the latter consistent with this conformation being easier to adopt an efficient stacking within the bulk crystal structure. Notably, while form II exhibits enhanced strength in both hydrogen bonds and aromatic stacking compared to form I, the latter demonstrates an overall greater contribution from weaker vdW interactions.^18^

### 3.2 Calculated Lattice Energies and Their Convergence

Lattice energies and their convergence for both forms I and II as a function of the limiting intermolecular distance provided previously^18^ indicated very similar lattice energies for both forms, consistent with the known low barrier for the interconversion of TFA,^17,18^ implying the ease of conformational change within the solution-state and solid-state environments underpinning the observed concomitant crystallization behavior of the material.

Analysis of the convergence behavior suggests that form II may generate stable initial nucleation clusters at smaller sizes, while form I stabilizes more efficiently at slightly larger cluster sizes, suggesting that supersaturation variation could potentially influence the polymorphic outcome by affecting the cluster size evolution.^18^ Intrinsic synthon energy decomposition further highlighted the main differences between these two forms. While the hydrogen bonding was similar in both forms (slightly stronger in II), form I exhibited stronger electrostatic contributions to its overall lattice energy, consistent with its more polarized twisted molecular conformation, whereas analysis of the form II structure showed a stronger vdW contribution, reflecting more effective π···π stacking enabled by its planar molecular conformation.^18^

### 3.3 Predicted Morphology and Surface Properties

The predicted morphology and the associated surface chemistry of the dominant crystal faces for the two forms of TFA are highlighted in Figure 3 together with the details of the intermolecular energies for each crystal habit face and their relative synthon contributions, given in Tables 1 and 2.

**Figure 3:**
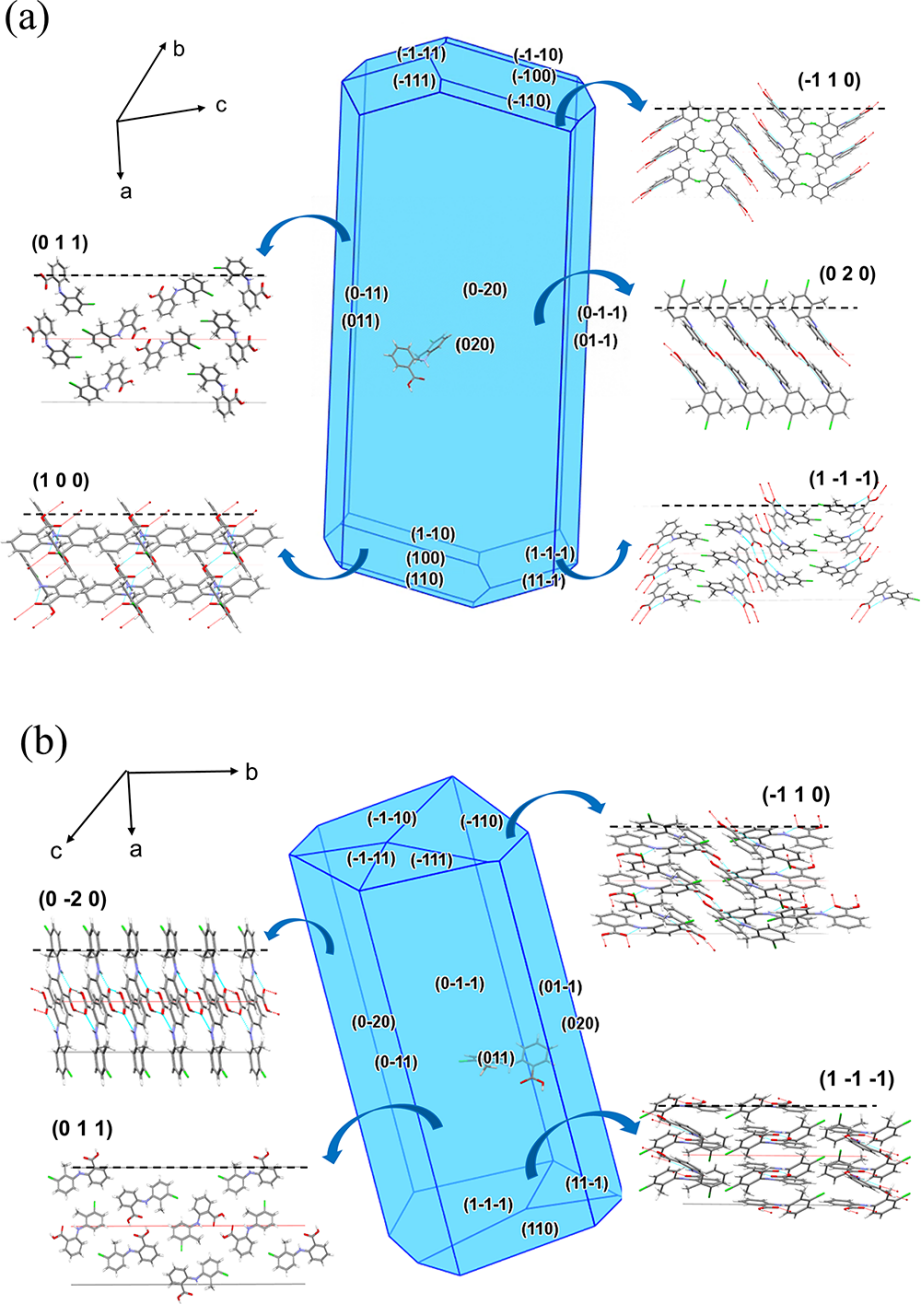
Predicted crystal morphologies for the morphologically important habit faces for the polymorphic forms I (a) and II (b), together with their associated surface chemistry.

**Table 1:**
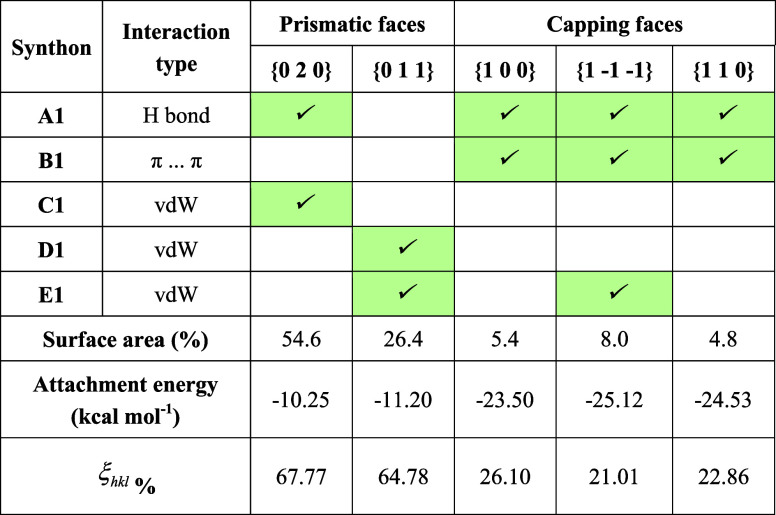
Synthon Analysis for the Dominant Crystal Faces of Form I for a Growth Unit^a^

a For each face, it is identified whether the top five extrinsic (growth promoting) synthons contribute to the attachment energy (highlighted in green).

**Table 2:**
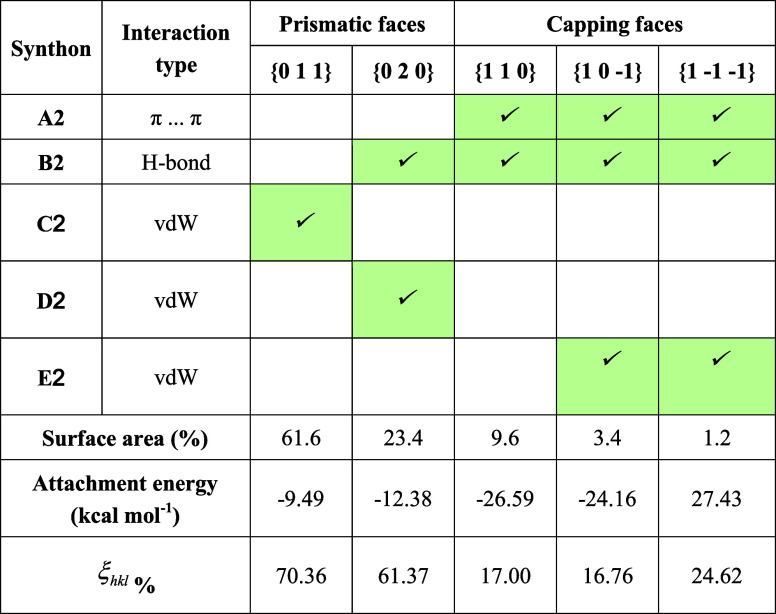
Synthon Analysis of the Dominant Crystal Faces of Form II for a Growth Unit

#### 3.3.1 Form I

As shown in Figure 3a, examination of the predicted morphology of form I reveals a long plate-like crystal morphology, which was characterized by {0 2 0}, {0 1 1}, {1 −1 −1}, and {1 1 0} crystallographically independent habit planes.

A detailed analysis of each dominant face in terms of surface attachment energies and associated surface chemistry is given in Tables 1 and S1 together with the intermolecular structures for the top five strongest synthons for each form, given in Figure S3. Analysis of the data reveals that the top two larger prismatic faces {0 2 0} and {0 1 1} exhibit a high degree of surface saturation (ξ_*hkl*_ = 67.77 and 64.78%, respectively), suggesting that lower numbers of unsaturated interactions would be available for growth, consistent with the low growth rate observed for these two faces. In contrast, a much lower degree of saturation (ξ_*hkl*_ = 26.10, 21.01, and 22.86%, respectively) was found for the capping faces {1 0 0}, {1 −1 −1}, and {1 1 0}, associated with the growth along the needle axis, indicating that sufficient unsaturated intermolecular interactions were available for solute attachment, consistent with the much faster growth along the needle axis.

Table 1 presents the extrinsic synthon analysis, highlighting the contributions of the top five strongest synthons in form I to each surface. The data suggest that the strong hydrogen bonds and π···π interactions mainly contribute to the growth of the two capping surfaces, consistent with the higher growth rates for these surfaces. The interactions at the surface can be visualized in Figure 3a, highlighting their aromatic stackings and exposed hydrogen bond donor and acceptor sites at the {1 0 0}, {1–1–1}, and {1 1 0} faces, which facilitate the formation of the strong synthons A1 and B1. In contrast, only weak vdW synthons contribute to the surface attachment energy of the prismatic habit face {0 1 1} surface. These do not contain any of the strongest growth-promoting extrinsic sythons and can be expected to be slow-growing, with the resultant larger surface area. For the larger prismatic face {2 0 0}, the hydrogen bond synthon A1 and weak vdW synthon C1 were found to contribute to its attachment energy. Visualization of the surface chemistry (Figure 3a) further suggests that only chlorine atoms and benzene rings are exposed at the prismatic crystal habit faces, consistent with their hydrophobic properties.

#### 3.3.2 Form II

The predicted morphology of form II, as illustrated in Figure 3b, was found to adopt an elongated rod-like shape, which was dominated by two large prismatic faces {0 1 1} and {0 2 0} and several capping {1 1 0}, {1 0 −1}, and {1 −1 −1} crystallographically independent crystal habit faces.

Examination of the anisotropy factors in Table 2 reveals that the {0 1 1} and {0 2 0} side faces, which have the top two large surface areas, adopt quite a high degree of surface saturation of intermolecular interactions (ξ_*hkl*_ = 70.36 and 61.37%, respectively). The low number of unsaturated interactions for growth suggests low attachment rates of solute molecules and hence low growth rates for these two faces. In contrast, the tiny capping faces {1 1 0}, {1 0 −1}, and {1 −1 −1} exhibit very lower degree of saturation, indicating stronger intermolecular binding along the needle axis.

Analysis of the detailed intermolecular interactions, as given in Table 2, revealed the capping faces {1 1 0}, {1 0 −1}, and {1 −1 −1} to be the only habit planes involving the strongest synthon A2. The second strongest synthon B2 was also found to contribute to the three capping faces, consistent with the strong intermolecular attachment for the solute at these three faces. In contrast, the larger {0 1 1} prismatic faces were not found to contain any contribution from these strong synthons with their growth being dependent only on the weak vdW interactions (synthons C2, D2, and E2), which correlates well with the predicted slow growth rate on these faces. Figure 3b displays the surface chemistry, demonstrating the hydrophobic properties of the {0 1 1} face with chlorine atoms and benzene rings being exposed on the surface. The capping face {−1 1 0} was found to exhibit obvious hydrophilic properties associated with the exposed hydrogen bond donor and acceptor sites, which could be expected to promote the formation of the strong hydrogen bonds.

### 3.4 Solvent-Dependent Crystal Morphologies

The experimentally observed crystal morphologies of TFA forms I and II crystallized from polar aprotic (acetonitrile), protic (methanol and ethanol), and nonpolar (toluene) solvents are given in Figure 4a,b, respectively. The data clearly show that both forms exhibit needle-like morphologies notably with higher aspect ratios than those predicted with subtle differences between the capping faces with form I exhibiting two asymmetrical tilted habit planes, while form II appears to be much flatter at the end of the needle-like crystals. A closer examination of the arrangements for the end faces using tilted incident angle microscopy is given in Figure 5, revealing there to be two tilted habit planes for form I compared to several tiny faces for form II. For form II, the actual capping faces observed fit quite well with the predicted morphology. However, the actual morphology of form I from the experimental observation is slightly different from the predicted one. While the larger face {−1 0 0} in form I was observed to exist in the experimental crystals (the larger top face), as predicted, the two smaller faces {−1 1 0} and {−1 −1 0} were not observed probably due to their relative faster growth rates in solvents. Faces {−1 1 1} and {−1 −1 1} seem to be replaced by one {−1 0 1} face. However, the predicted morphology generally agrees with the experimental observations (Figure 4a), although the latter is found to exhibit a higher aspect ratio, consistent with differential solvent-mediated growth inhibition.

**Figure 4:**
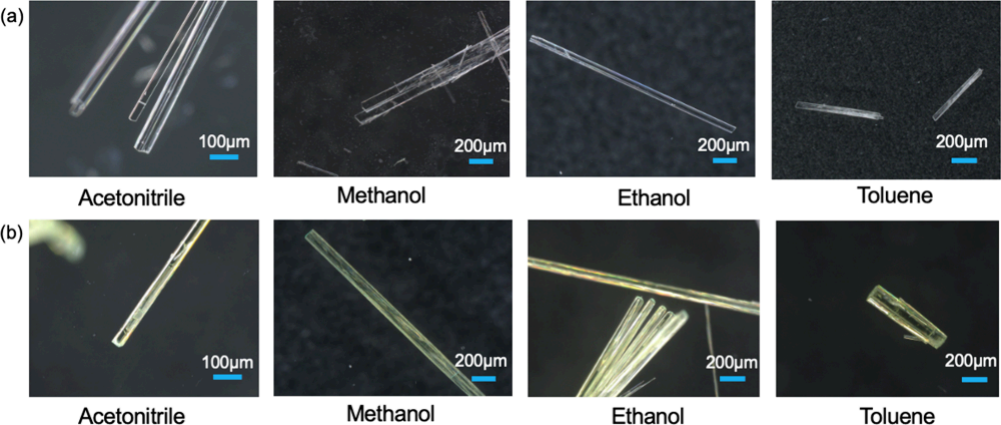
Optical microscopic images focused on the observed morphologies as a function of the crystallization solvent for forms I (a) and II (b).

**Figure 5:**
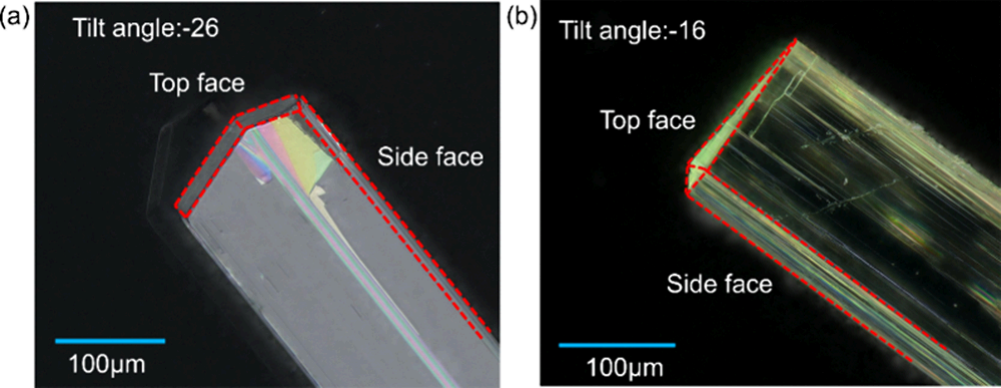
Microscopic images focused on the top face and side face for forms I (a) and II (b) with tilt angles being −26 and −16°, respectively.

Form I crystals were found to display a consistent plate-like morphology for all of the solvents examined, with polar protic solvents such as ethanol yielding higher aspect ratios when compared to the apolar toluene. Such variations would be consistent with the solvent-specific intermolecular interactions at the growth interface. Polar solvents would be likely to bind to the {0 2 0} prismatic faces and the capping faces by forming solvation hydrogen bonds. Such a binding was expected to compete with the formation of the solute–solute extrinsic synthon A1 and hence slow desolvation and inhibit the growth on not only the large prismatic faces but also the three capping faces.

However, the growth of capping faces involves not only hydrogen bond formation but also vdW interactions associated with aromatic stackings, which is reflected in the observation that the crystals obtained from toluene solutions showed much alower aspect ratios compared to those obtained from the polar solvents. The latter could be attributed to the formation of strong solute/solvent π ... π interactions at the capping faces, which decreases desolvation due to enhanced surface/solvent interactions and hence effectively decreases the growth rate along the needle axis directions.

The observed solvent-dependent morphology of form II was found in general to mirror that for form I, although with a stronger solvent effect on the aspect ratio of form II than that for form I (Table 3). Form II crystals exhibited higher aspect ratios in polar protic solvents than in toluene, following the same inhibition mechanisms in form I, i.e., forming hydrogen bonds by polar solvents on key faces, while enhancing the inhibition by toluene via π···π interactions of synthon A2 and hence at the capping faces.

**Table 3:** Comparative Growth Rates for Forms I and II in Ethanolic Solutions at a Relative Solution Supersaturation of 0.3 at 20 °C

	form I		form II	
	long axis	short axis	long axis	short axis
lattice plane	(1 0 0)/(−1 0 0)	(0 1 1)/(0 −1 −1)	(1 1 0)/(−1 1 0)	(0 2 0)/(0 −2 0)
growth rate (μm/s)	0.124	0.002	0.194	0.003
aspect ratio	22.23		31.26	

### 3.5 Comparison between Growth Rates of the Two Forms

Examination of the growth rates for form I and II crystals for both the capping and prismatic faces in ethanolic solutions at the same supersaturation, as given in Table 3, reveal them to be of the same order of magnitude as that previously reported for their growth rates in isopropanol solution.^31^ As expected, capping faces were found to grow much faster than the prismatic face for both forms I and II, consistent with their observed needle-like morphologies and large aspect ratios. Overall, the growth rate of form II was found to be higher than that of form I (Table 3), especially along the needle axis direction, consistent with the lower predicted surface saturation for the capping face in form II.

Since form II transforms readily to form I in ethanol, it was not found to be feasible to record a wide range of its growth rates as a function of supersaturation. Notably, form II crytsals were observed to transform to form I during the growth rate measurement (typically toward the end of the experiment). Therefore, only the data collected prior to the transformation were used to determine the growth rate of form II. In this study, only growth rates at σ = 0.3 (green points in Figure 6) were collected for form II.

**Figure 6:**
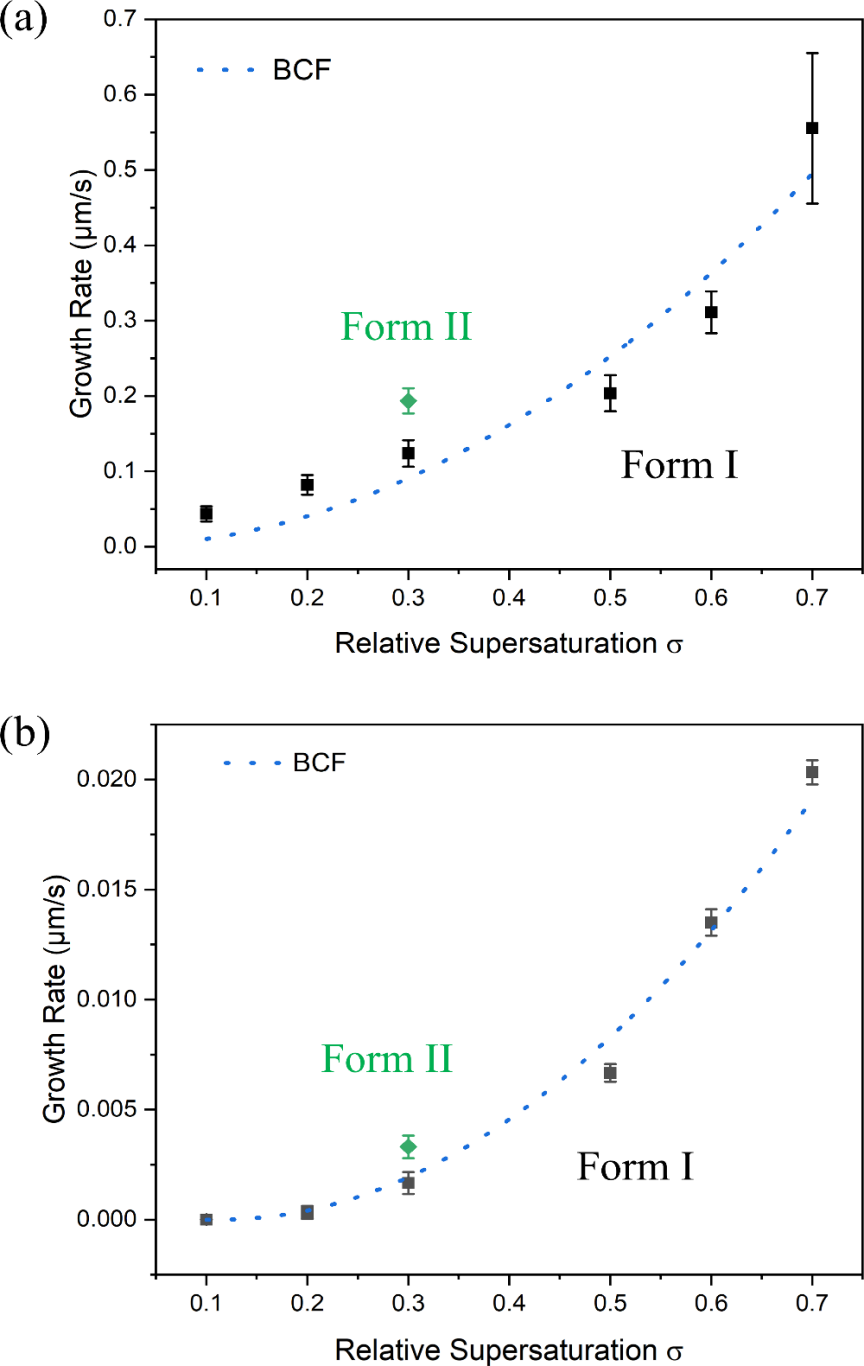
Growth rate experimental data from ethanolic solutions fitted to the BCF models for the (1 0 0) capping face (a) and the prismatic (011) face (b) for forms I, with the growth rate of form II at σ = 0.3 given for comparison.

### 3.6 Supersaturation-Dependent Growth Rates and Growth Kinetics

The growth kinetics of TFA form I in ethanolic solutions as a function of supersaturation (σ = 0.1–0.7) are given in Figure 6, with facet-specific growth rate kinetic parameters being detailed in Table 4. The associated original images as recorded during the growth measurements are given in Figures S4–S6 (SI) together with the fitting method for the determination of crystal growth rates being detailed in Figures S1 and S2 (SI).

**Table 4:** Crystal Growth Kinetic Parameters for Form I Obtained from the Best Fitting of Experimental Growth Data with the BCF Models (eqs 1 and 5)­^a^

fitting model	parameters	capping faces (1 0 0)/(−1 0 0)	prismatic faces (0 1 1)/(0 −1 −1)
BCF	$\frac{1}{k_{\mathrm{MT}}^{\prime }}$	1.03 × 10^7^	4.88 × 10^6^
	$\frac{1}{k_{\mathrm{GS}}}$	4.89 × 10^6^	1.67 × 10^7^
	σ_crit_	0.0007	0.11
	*A*_2_	30.42	71.49
	*R* ^2^	0.92	0.93

a Note that the values of *k*_*GS*_ were calculated using the σ = 0.4 (the average value studied in this work).

As expected, the growth rate was found to increase with supersaturation (Figure 6) with the capping faces (1 0 0)/(−1 0 0) being found to grow much faster than those of the prismatic faces (0 1 1)/(0 −1 −1) for all supersaturations, with typical growth rates of the former being about 20–70 times higher than those of the latter. Notably, no growth was observed to occur on the prismatic faces at supersaturations lower than 0.11 (referred to as a critical supersaturation σ_crit_ = 0.11), consistent with a “dead zone” for growth, associated with the need for a critical supersaturation threshold for initiating the lateral growth process. This observation would be consistent with the larger 2D surface nucleation cluster sizes needed for growth at these low supersaturations.

Modeling the experimental data against the three growth kinetics models and including the growth resistance of both MT and GS have been carried out with the “dead zone” for growth (σ_crit_ = 0.11) for the faces (0 1 1)/(0 −1 −1) being also included by subtracting σ_crit_ from σ.^5,32^ Figure 6 shows the fit of the BCF model to the experimental data in ethanol for the (1 0 0) and (0 1 1) faces. All relevant parameters obtained through this model are presented in Table 4, while the other model fits are given in Table S3 (SI).

For the (1 0 0) capping face, the best fittings for TFA growing from ethanol were obtained from both the power law (*R* ^*2*^ = 0.93) and the BCF mechanism (*R* ^*2*^ = 0.92). Notably, the derived *r* value (2.28) in the power law is close to 2, which is also consistent with the BCF mechanism. The derived MT resistance value was found to be much higher than the GS resistance value, indicating that the growth at the capping (1 0 0) face is mainly hindered by the diffusion of TFA in the bulk solution.

For the (0 1 1) prismatic face, the BCF model still shows quite good fitting results with *R* ^*2*^ being 0.93. Although a higher *R* ^*2*^ value (0.99) was achieved in the power low model, the derived *r* value (*r* = 2) for this model indicates that the growth mechanism is also consistent with the BCF mechanism. In contrast to the (1 0 0) face, the GS resistance was found to be higher than the MT resistance for the (0 1 1) face, highlighting that the integration of growth units is the rate-limiting step for the prismatic faces. This correlates well with the difficulty in integration of solute into the (1 0 0) surface caused by the high degree of surface saturation (ξ_*hkl*_ > 64%) of the strong intermolecular synthons (Table 2).

### 3.7 Discussion

As shown in Table 5, a comparative analysis of the growth rates in the literature as a function of the relative solution supersaturation (σ) for individual crystal habit faces of some organic compounds in various solvents^33^ reveals the growth rates for TFA forms I and II obtained in this work to be broadly similar to those measured for similar organic systems.^4,5,32–38^ The {1 0 0} capping and {0 1 1} prismatic faces of form I were found to exhibit growth rate ranges similar to those of the {1 −1 0} capping and {1 1 0} prismatic faces of methyl stearate,^5^ with the latter being measured in n-dodecane, kerosene, and toluene at a lower range of solution supersaturations of 0.04–0.39, compared to 0.10–0.70 for TFA ethanol systems.

**Table 5:** Comparative Published Growth Rates for Individual Crystal Habit Faces of Some Organic Crystalline Systems

compounds	σ	range of growth rates (μm/s)	references
stearic acid (B and C polymorphs) {110} faces in butanone	0.01–0.30	0.00–2.80	^35^
stearic acid (B polymorph) {110} faces in decane	0.01–0.40	0.00–0.40	^34^
dotriacontane (C_32_H_66_) {110} faces in m-xylene	1.00–1.50	1.00–3.00	^36^
ibuprofen {001)} and {011} faces in ethanol/water, ethyl acetate, acetonitrile, and toluene	0.55–1.3	0.04–2.02	^4^
n-docosane {010}, {112}, {102}, and other non-indexed faces in n-dodecane	0.01–0.05	0.51–9.85	^32^
methyl stearate {110} and {1–10} faces in			^5^
n-dodecane	0.30–0.39	0.09–1.13	
kerosene	0.45–0.52	0.01–0.35	
toluene	0.04–0.08	0.02–0.37	
L-glutamic acid (β form) in water			
{101} faces	0.28–1.21	0.46–4.01	^37^
{10–1} faces	0.28–1.21	0.52–4.13	^37^
{021} faces	0.28–1.21	0.01–0.44	^37^
{010} faces	1.05	0.21	^38^
para aminobenzoic acid in ethanol			^33,39^
{01–1} faces	0.10–0.20	0.16–3.60	
{10–1} faces	0.10–0.20	0.15–0.30	
TFA in ethanol			this work
form I {100} and {011} faces	0.10–0.70	0.00–0.56	
form II {110} and {020} faces	0.3	0.194 and 0.003	

Table 6 draws together recent work on TFA crystallization research and presents a summary overview from the assessment of the solubility, nucleation kinetics, and polymorph screening of the material with the data presented here.^17,18^

**Table 6:** Summary of Parameters Obtained through the Combined Assessment of Solubility, Nucleation, Polymorphic Forms, Morphology, and Growth Kinetics of TFA in Ethanol

		ethanol				methanol	acetonitrile	toluene
solution properties	solubility	higher				intermediate	lowest	lower
	diffusion coefficient (10^–10^ m^2^ s^–1^)	2.55–6.5				6.33–12.88	7.23–19.78	6.72–12.38
nucleation of form II	ease of nucleation	difficult				intermediate	easiest	easier
	range σ_*crit*_	1.05–1.99				0.81–1.54	0.29–0.50	0.71–0.81
	nucleation rate	7.50 × 10^8^–9.74 × 10^9^				7.38 × 10^9^–6.14 × 10^10^	1.08 × 10^13^–2.48 × 10^13^	4.77 × 10^10^–6.39 × 10^10^
	γ_eff_ (mJ/m^2^)	4.27–5.74				3.00–5.30	1.64–2.73	2.80–3.93
conclusions	The solubility and nucleation are solvent-dependent and highly correlated with each other, which can be interpreted by the strength of solvent–solute interactions, where a stronger solvation interaction will lead to a higher solubility and also the difficulty of desolvation during nucleation. Higher solubility is associated with higher viscosity, which limits the molecular diffusion and in turn increases the difficulty of nucleation.							
polymorph selection	preferred polymorph	form II				form II	form II	form II
conclusions	The nucleation of TFA polymorphic forms exhibits a stochastic nature, which can be interpreted by close similarities in both molecular and solid-state stabilities. The metastable form II has lower conformational deformation energy and tends to be preferred in most of the conditions while the formation of the stable form I requires higher solute concentrations. Toluene tends to reduce the probability of obtaining form I.							
morphology	form I	needle				needle	needle	needle (low aspect ratio)
	aspect ratio	∼28				∼23	∼21	11–15
	form II	needle				needle	needle	needle (low aspect ratio)
	aspect ratio	∼32				∼24	∼23	4–6
crystal growth		form I		form II				
	σ	0.1–0.7		0.3				
		(1 0 0)	(0 1 1)	(1 1 0)	(0 2 0)			
	*G* (μm/s)	0.044–0.555	0–0.020	0.194	0.003			
	growth mechanism	BCF	BCF					
	rate-limiting step	mass transfer in bulk solution	integration of solute at interface					
conclusions	Forms I and II both exhibit a long needle-like shape in all of the solvents, as predicted. A lower aspect ratio was found in the apolar toluene compared to other solvents due to the intermolecular interactions between toluene and the capping face, which slow desolvation and hinder the integration of solute into it. The facet-specific growth rate of form I suggests much higher growth rates for the capping faces than the prismatic faces, which is consistent with the observed needle-like shape. The growth rate of form II was found to be higher in both directions. Kinetics analysis of form I revealed that the growth at (1 0 0) and (0 1 1) faces proceeds through the BCF mechanism.							

Examination of the solution properties reveals the higher viscosity as well as the low diffusion coefficient of TFA in ethanol, consistent with the mass transfer within the bulk solution playing important roles in the crystallization of TFA from ethanol solution. Analysis of both the nucleation and growth kinetics supports this conclusion notably, with the solute diffusion being the rate-limiting step in directing the nucleation rate and the growth rate on the fast-growing {100} capping faces reflecting the fact that attachment energies of these faces are high, and thus, the integration of the solute into the surface can be expected to be quite easy. Comparatively, the growth rate along the {011} prismatic faces was found to be dependent on the interface kinetics due to the lower attachment energies and their less active binding sites on this surface to form expanding interactions with the solute based on the surface chemistry analysis.

It can thus be concluded that the polymorphic crystallization of TFA is influenced by both the nucleation and growth process. In practical terms, the metastable form II tends to be always the most preferred form in all crystallization conditions examined here, with form I being more difficult to obtain. The latter reflects the greater conformation change from planar- to twisted-like conformation of form I in its solution-mediated crystallization pathway from its solvated molecular state through nucleation to the solid state. The observation of a higher growth rate of form II with respect to form I also supports the easier formation of form II, suggesting that both nucleation and growth are promoted for the formation of form II in the solution state.

Solvents have obvious effects on directing the nucleation rate, which were found to have less of an effect on the crystal morphology except for toluene. The crystals of TFA adopt a long needle-like shape for both forms in all of the solvents. The only difference lies on the aspect ratio in which polar protic solvents will produce a longer aspect ratio than the aprotic toluene solvents, which is due to the aromatic interactions between toluene and the capping faces. Solvents are usually considered to affect the growth kinetics significantly. However, only the growth kinetics of TFA form I from ethanol solution has been investigated, and so further work is needed regarding the growth kinetic data of TFA from more solution systems and importantly for form II in order to gain a deeper understanding of the growth mechanism and associated polymorph control of TFA in various solution environment.

## 4 Conclusions

A detailed analysis of the crystal morphology for the two forms of TFA was carried out, encompassing both computational molecular-scale modeling and experimental approaches. The crystal morphology predicted reveals good agreement with the experimental data, highlighting the long needle-like morphology of TFA for both forms I and II. Subtle differences in the arrangement of the capping faces between the two forms have been characterized in terms of the bulk crystal chemistry and surface chemistry of the material. Solvent effects on the morphology were also investigated using both polar and apolar solvents, with the main effect being the changes in the aspect ratio, which is further rationalized by examining the interactions between the solvent and the growing crystal surfaces. The growth rates of the stable form I were found to be less than that of the metastable form II in ethanolic solutions, with obviously slower growth rates being observed on the side faces compared to the capping faces, which is consistent with their observed needle-like morphology. Analysis of the growth rates versus supersaturation fits well with the BCF mechanism for both capping and side faces, with the growth rates of the side faces being limited by the integration of the growth unit onto the surface, consistent with their high synthon surface saturation in contrast to the faster-growing capping faces where mass transfer diffusion in solution plays a stronger role, reflecting the lower synthon saturation of these faster-growing surfaces. A comparison with the growth kinetics of form II was not found to be feasible in this study due to its instability to transformation to form I. Further work is still needed in this respect and also regarding solvent-specific kinetics.

Overall, this integrated work deepens the mechanistic understanding of crystal growth of TFA and pharmaceutical compounds in general providing helpful insights into the behavior of a concomitant polymorphic system and also toward providing a framework for modulating the crystal morphology as part of the digital design of crystallization processes.

## Supplementary Material



## Notes

Fitting method for determining growth rates; synthon structures in TFA crystals; morphology prediction of TFA; typical sequence of crystal images; facet growth rates of TFA form I; crystal growth kinetics parameters (PDF)

## List of Symbols and Abbreviations

*A*_1_: Thermodynamic parameters in the B&S model
*A*_2_: Thermodynamic parameters in the BCF model
*A*_*ij*_, *B*_*ij*_, *C*_*ij*_, *D*_*ij*_: Atom–atom force field parameters for atoms *i* and *j* in the first and second molecules, respectively
*D*: Dielectric parameter
*d*_*hkl*_: Growth layer thickness
*E*: Intermolecular potential energy
*E*_cr_: Lattice energy
*E*_sl_: Slice energy
*E*_att_: Attachment energy
*G*: Facet growth rate
*hkl*: Crystal face index
*k*_G_: Growth rate constant
*q*_*i*_, *q*_*j*_: Atomic point charges
*r*: Growth exponent
*r*_*ij*_: Central distance between atoms *i* and *j*
*x*: Solution concentration
*x*_e_: Mole fraction solubility
$\frac{1}{k_{\mathrm{GS}}}$: Resistance of integration of growth units at the surface
$\frac{1}{k_{\mathrm{MT}}^{\prime }}$: Resistance of mass transfer in the bulk solution
σ: Relative supersaturation
ξ_*hkl*_: Surface anisotropy factor
GS: Integration of growth units at the surface
MT: Mass transfer in the bulk solution
TFA: Tolfenamic acid
vdW: van der Waals
