# Supplementary material for: Crystal Morphology and Associated Face-Specific Growth Kinetics of Tolfenamic Acid as a Function of Its Solution Crystallization Environment
Source: Cryst Growth Des. 2025 Oct 21;25(21):9322–32. doi: 10.1021/acs.cgd.5c01129 (PMC12593351; doi:10.1021/acs.cgd.5c01129)
Supplement: Supplementary file 1 [file cg5c01129_si_001.pdf]

Supporting Information

Crystal Morphology and Associated Face-Specific Growth Kinetics of Tolfenamic Acid as a Function of its Solution Crystallisation Environment §

Yu Liu,[1, 2, 3] Cai Y. Ma,[2] Junbo Gong,[3] and Kevin J. Roberts[2,*]

[1] School of Chemical Engineering, Shenyang University of Chemical Technology, Shenyang, China

[2] School of Chemical and Process Engineering, University of Leeds, Woodhouse Lane, Leeds LS2 9JT, UK

[3] State Key Laboratory of Chemical Engineering, Tianjin University, Tianjin, China

* Corresponding author: K.J.Roberts@leeds.ac.uk

§ Special Issue of Crystal Growth & Design: Design of Crystals via Crystallization Processes

The supplementary material supports the main manuscript by providing further details of the following: **Figures S1 – S2** presents the typical fitting method for the determination of crystal growth rates. **Figure S3** presents the structures of the top five strongest synthons of TFA form I, and form II. **Figures S4 – S6** gives the typical original crystal images captured during the growth rate measurements. **Table S1** presents Calculated attachment energies, slice energies and degree of surface saturation for the dominant crystal faces for forms I and II. **Table S2** gives facet growth rates for form I with different supersaturations **Table S3** gives the crystal growth kinetics parameters obtained from the different fitting models.

S1. Determination of the Growth Rates

The growth rates were determined through liner fitting of the original measurement data as given in **Figure S1** with the gradient of the fitting line being used as the determined growth rate.

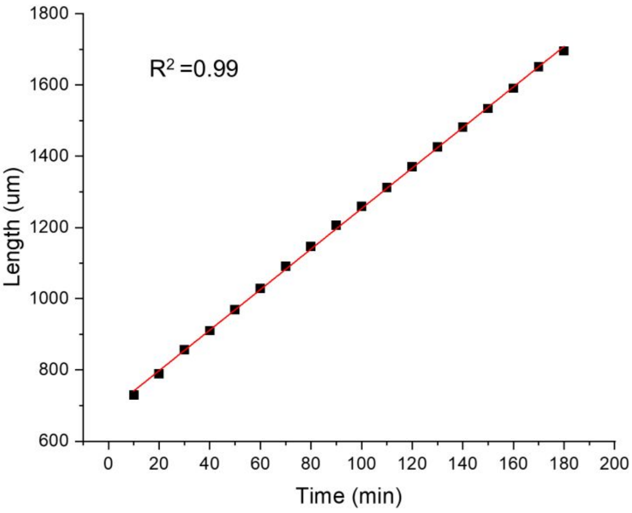

Figure S1. Determination of growth rate through plotting measured length as a function of relative time.

In some cases, there are two stages shown in the length vs. time plot, as given in **Figure S2**. We only used the data at the beginning of the growth. For example, the blue line fits the first 70 min of data.

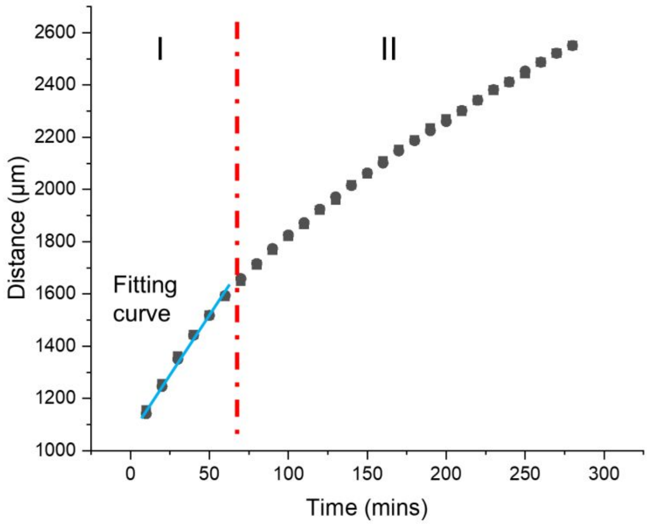

Figure S2. Determination of growth rate through plotting measured length as a function of relative time with two stages (I and II) showing in the plot. Only the first 70 min of data was used in this case for growth rate determination (blue fitting line).

S2. Synthon Structures in TFA

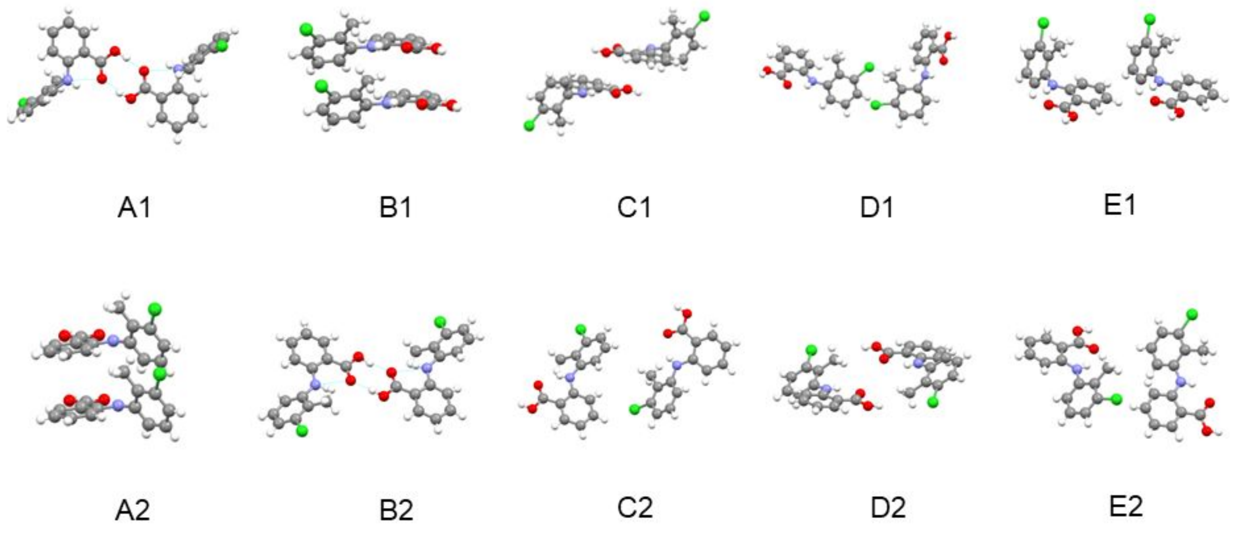

Figure S3. The intermolecular structural arrangements for dimer pairs of the top five strongest synthons of TFA form I (A1 - E1) and form II (A2 - E2).

S3. Morphology Prediction of TFA

Table S1. Calculated attachment energies, slice energies and degree of surface saturation for the dominant crystal faces for forms I and II.

Form	*hkl*	Multiplicity	Surface area (%)	Slice energy (kcal mol^{-1})	Attachment energy (kcal mol^{-1})	ξ_{hkl} %
**I**	0 2 0	2	54.6	-21.55	-10.25	67.77
	0 1 1	4	26.4	-20.60	-11.20	64.78
	1 0 0	2	5.4	-8.30	-23.50	26.10
	1 -1 -1	4	8	-6.68	-25.12	21.01
	1 1 0	4	4.8	-7.27	-24.53	22.86
**II**	0 1 1	4	61.6	-22.55	-9.49	70.36
	0 2 0	2	23.4	-19.67	-12.38	61.37
	1 1 0	4	9.6	-5.45	-26.59	17.00
	1 0 -1	2	3.4	-5.37	-26.68	16.76
	1 -1 -1	4	1.2	-7.89	-24.16	24.62
	1 0 1	2	0.2	-4.62	-27.43	14.41

S4. Typical Sequence of Crystal Images

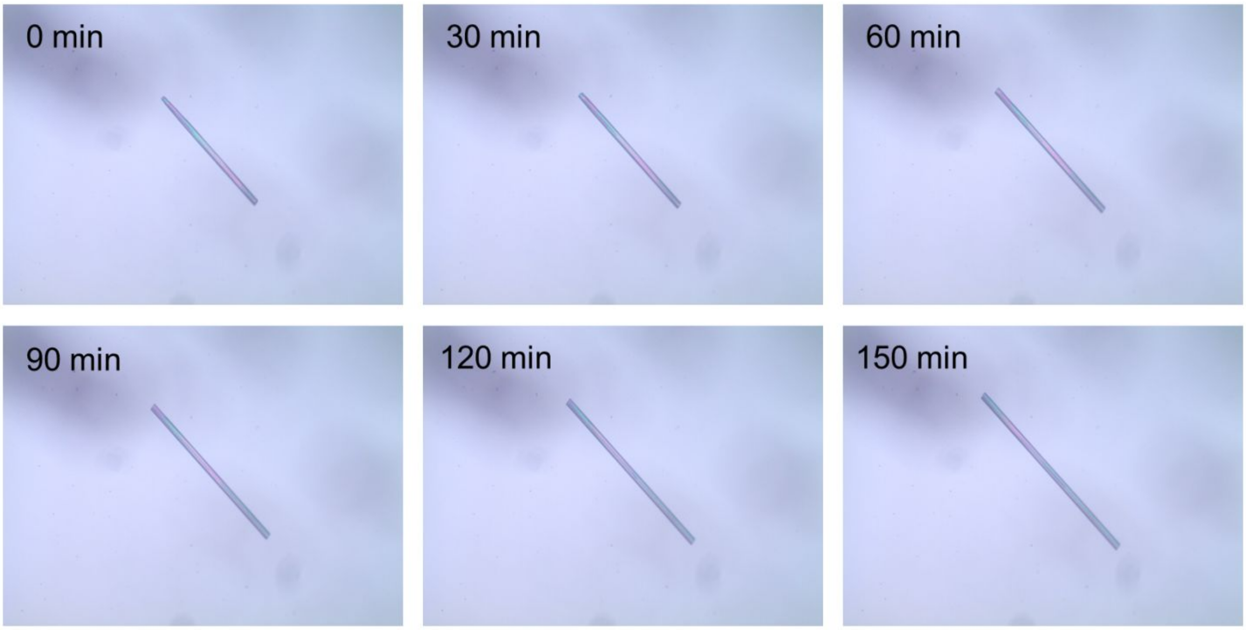

Figure S4. Typical sequence of images of TFA form I growing in ethanol with time at relative supersaturation of 0.1.

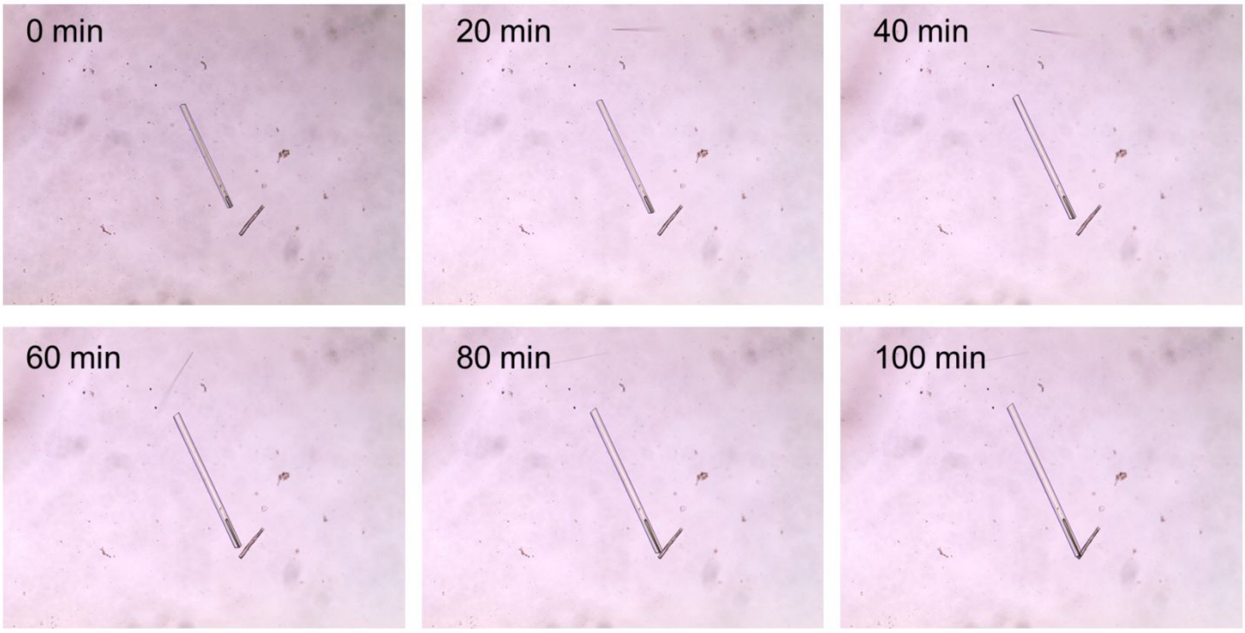

Figure S5. Typical sequence of images of TFA form I growing in ethanol with time at relative supersaturation of 0.3.

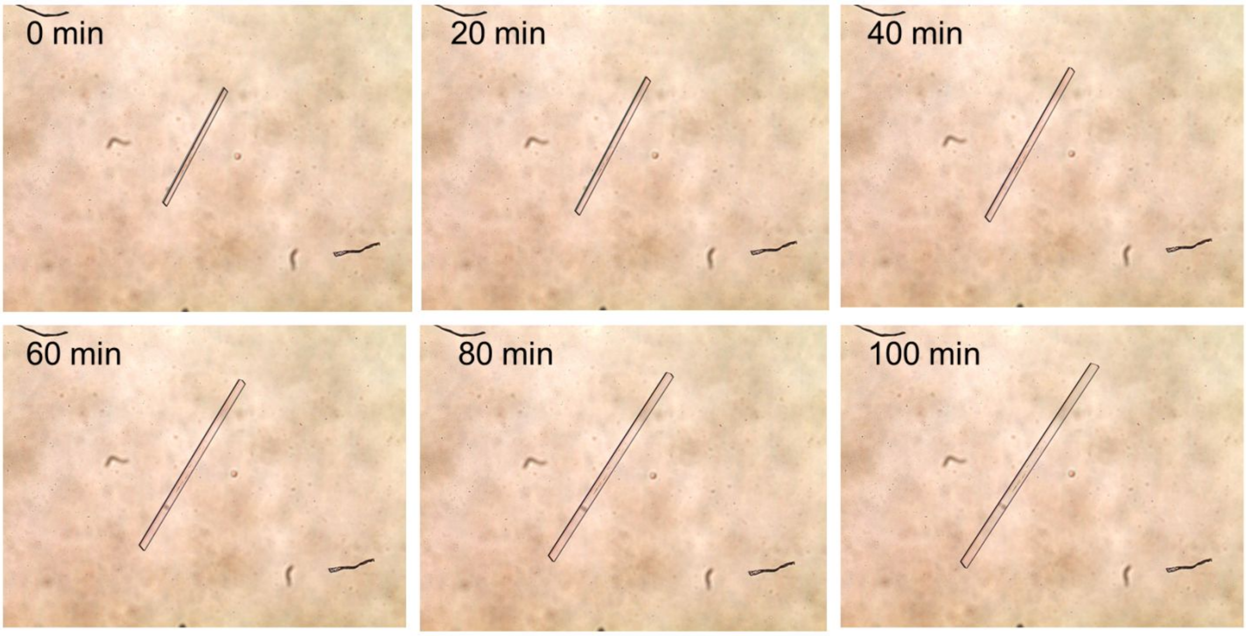

Figure S6. Typical sequence of images of TFA form I growing in ethanol with time at relative supersaturation of 0.6.

S5. Facet Growth Rates of Form I

Table S2. Facet growth rates of (1 0 0) capping and (0 1 1) side faces with different supersaturations for form I.

Relative supersaturation	Growth Rate (μm/s)	
	(1 0 0) / (-1 0 0)	(0 1 1) / (0 -1 -1)
0.1	0.0435	0
0.2	0.0818	0.0003
0.3	0.1237	0.0017
0.5	0.2034	0.0067
0.6	0.3109	0.0135
0.7	0.5554	0.0203

S6. Crystal Growth Kinetics Parameters

Table S3. Crystal growth kinetics parameters obtained from the best fitting of experimental growth data with the different models (Eqs. 1-4)[a].

<table>
<tr><th>Fitting Model</th><th>Parameters</th><th>Capping Faces (1 0 0) / (-1 0 0)</th><th>Prismatic Faces (0 1 1) / (0 -1 -1)</th></tr>
<tr><td rowspan="4">Power law</td><td>$\frac{1}{k'_{MT}}$</td><td>4.22×10^{6}</td><td>4.47×10^{6}</td></tr>
<tr><td>$\frac{1}{k_{GS}}$</td><td>2.76×10^{6}</td><td>9.53×10^{7}</td></tr>
<tr><td>r</td><td>2.28</td><td>1.99</td></tr>
<tr><td>R^2</td><td>0.93</td><td>0.99</td></tr>
<tr><td rowspan="4">B&S</td><td>$\frac{1}{k'_{MT}}$</td><td rowspan="4">No reasonable fit</td><td rowspan="4">No reasonable fit</td></tr>
<tr><td>$\frac{1}{k_{GS}}$</td></tr>
<tr><td>A_1</td></tr>
<tr><td>R^2</td></tr>
</table>

[a] Note that the values of k_{GS} were calculated using the σ = 0.4 (the average value studied in this work).
